# Cannabidiol lymphatic transport after oral administration assessed using a novel thoracic lymph duct cannulated conscious pig model

**DOI:** 10.1080/10717544.2025.2608913

**Published:** 2025-12-29

**Authors:** Vitalii Rizov, Peter Lukáč, Mikuláš Mlček, Petr Kozlík, Tomáš Křížek, Petr Jelínek, Petr Šodek, Michaela Sklenárová, Viktória Paulusová, Olesia Symkanych, Daniel Stránský, Anežka Klouček, Miroslav Šoóš, Martin Šíma, Tomáš Grus, Ondřej Slanař, Pavel Ryšánek

**Affiliations:** a Deparment of Cardiovascular Surgery, First Faculty of Medicine, Charles University and General University Hospital in Prague, Prague, Czech Republic; b Deparment of Cardiac Surgery, Masaryk Hospital in Ústí nad Labem, Ústí nad Labem, Czech Republic; c Institute of Physiology, First Faculty of Medicine, Charles University in Prague, Prague, Czech Republic; d Deparment of Analytical Chemistry, Faculty of Science, Charles University in Prague, Prague, Czech Republic; e Department of Chemical Engineering, Faculty of Chemical Engineering, University of Chemistry and Technology, Prague, Czech Republic; f Children's Cardiac Center, Second Faculty of Medicine, Charles University and Motol University Hospital, Prague, Czech Republic; g Institute of Pharmacology, First Faculty of Medicine, General University Hospital in Prague, Charles University, Prague, Czech Republic

**Keywords:** Cannabinoids, pharmacokinetics, oral bioavailability, interspecies comparison, lymph sampling, animal model development

## Abstract

Lymphatic transport of drugs after oral administration is an important physiological process in highly lipophilic compounds, such as cannabidiol (CBD). The majority of lymphatic transport studies have been historically conducted in anesthetized rats. However, this animal model differs significantly from the humans regarding both anatomical and physiological features. The aim of this study was therefore to develop a novel animal model using pigs and to provide an interspecies comparison for the lymphatic transport of CBD. The thoracic lymph duct was cannulated via thoracotomy in three pigs and lymph and blood were sampled from conscious animals to assess the lymphatic transport parameters and basic pharmacokinetic parameters of CBD administered in two distinct drug formulations (sesame oil-based solution and nanoemulsion) using a two-period cross-over study design. The mean ± SD oral bioavailability (F) was 6.1 ± 0.9% for the oil solution and 9.2 ± 6.6% for the nanoemulsion. The relative bioavailability via lymph (F_RL_), i.e. the percentage of the systemically available drug that has been transported through the mesenteric lymph, was 20 ± 10% and 11 ± 13%, respectively. Whereas the F_RL_ for the oil solution was 2.3-fold lower in pigs compared to rats, the F_RL_ for the nanoemulsion was almost identical for both species. In conclusion, the lymphatic transport of CBD plays an important role after its oral administration. The particular parameters differed significantly between the rodent and higher non-rodent species. The use of higher species models is therefore warranted for the lymphatic transport assessment in settings close to humans.

## Introduction

Intestinal lymphatic transport after oral administration is an important route of absorption and distribution of highly lipophilic drugs (log *P* > 5) (Porter et al. 2007; Rysanek et al. 2020). After solubilization in the gastrointestinal tract, these compounds are absorbed into enterocytes, the intestinal epithelial cells, where they are incorporated into chylomicrons. Chylomicrons are large lipoprotein particles produced specifically in the intestines that are naturally involved in the transport of highly lipophilic molecules. Their size/diameter is too large to pass into the intestinal blood capillaries. Therefore, they take an alternative route via the intestinal lymphatic system. Chylomicrons enter the lacteals (lymph capillaries) and are transported through a network of lymphatic vessels and mesenteric lymph nodes until they reach the thoracic duct, where the intestinal lymph combines with the lymph from the lower parts of the body. All the lymph eventually enters the systemic blood circulation at the confluence of the thoracic duct with the jugular and subclavian veins.

The transport through the intestinal lymphatic system can have a major impact on the drug pharmacokinetics. It can increase the absolute oral bioavailability because the lymph represents an additional gateway into the systemic circulation besides the standard transport through the portal vein. Another mechanism for increasing oral bioavailability involves avoiding first-pass metabolism in the liver. The mesenteric lymph is a dominant (sometimes even exclusive) source of systemically available drugs for compounds with a high extraction ratio in the liver (Shackleford et al. 2003; White et al. 2009).

The precise measurement of the lymphatic transport of orally administered compounds is a challenging task. This method requires lymph duct cannulation and lymph sampling in animal models. Several such models have been introduced in the last eighty years with the anesthetized mesenteric lymph duct-cannulated rat model being the first and so far also the most frequently used model (Bollman et al. 1948; Trevaskis et al. 2015). Another well-established model is a thoracic lymph duct-cannulated conscious dog (Khoo et al. 2001; Khoo et al. 2003). Scarce lymphatic transport studies have also been reported in mice (Trevaskis et al. 2013), cats (Forth et al. 1969), and pigs (White et al. 1991). There are even some historical reports of lymphatic transport measurement in men (Blomstrand and Forsgren 1968; Horst et al. 1976). Nevertheless, these studies were performed in patients with thoracic lymph duct catheters inserted for therapeutic reasons (e.g. immunosuppression before the era of modern immunosuppressive drugs). Since there is no standard indication for thoracic lymph duct cannulation in the modern medicine, there is currently no ethically admissible method of lymphatic transport measurement in men.

The anesthetized mesenteric lymph duct-cannulated rat model is relatively inexpensive and available. It requires delicate surgical skills, but once mastered, it is an effective method for generating several lymphatic drug profiles in one working day (authors' experience) (Salamunova et al. 2023; Pozniak et al. 2024). The drawbacks are anesthesia, which usually inhibits the total amount of drug absorbed (although the ratio between lymphatic and standard absorption through the portal vein remains constant) (Dahan et al. 2007), the need for intraduodenal dosing of liquid drug formulations with no possibility of standard human capsules or tablets, and strong differences in the anatomy and physiology of the gastrointestinal tract in rats compared to human (smaller volumina, smaller amount of gastric and intestinal fluid, absence of gall bladder, etc.) (Higashiyama et al. 2018).

These disadvantages have initiated the development of an animal model using higher species, namely a thoracic lymph duct cannulated conscious dog. After some pioneering works describing the first lymph duct cannulations in dogs for other reasons in the 1960s and 1970s (Rajpal and Kirkpatrick 1972), the first use of dogs for lymphatic transport measurement of drugs occurred around the millennium change (Khoo et al. 2001). The inclusion of this new method sparked a series of high-quality, well-designed lymphatic transport studies investigating standard human drug formulations, the effect of food and interspecies comparisons (Khoo et al. 2003; Trevaskis et al. 2009, 2013).

Although very informative and generally suitable for preclinical pharmacokinetics testing in the course of new drug development, dog models still have limitations precluding an easy data extrapolation towards humans in some cases. The gastric pH in dogs is more variable and usually higher compared to human (Lui et al. 1986). Furthermore, some important metabolic enzymes, such as cytochromes of the 2C9 and 2C19 subfamilies and N-acetyltransferases NAT1 and NAT2, are not expressed in dogs (Dalgaard 2015). Neither of these limitations are present in pigs. The gastric pH in pigs is comparable to that in humans and 2C9, 2C19, NAT1, and NAT1 enzymes (among others) are expressed. There is one more enzymatic difference that makes the pig a better model compared to dog specifically for the cannabidiol (CBD) pharmacokinetics testing. In dog, the CBD metabolism occurs largely via the cytochrome P450-CYP1 family, producing much less 7-COOH-CBD compared to pigs (and humans), where metabolism over the CYP3 family is dominant (Court et al. 2024). The pig is used less frequently for preclinical pharmacokinetics testing and no robust model for lymphatic transport measurement is currently available.

Cannabidiol (CBD) is an important non-psychoactive cannabinoid present naturally in cannabis and is used in several registered drug products (e.g. Epidyolex®, Jazz Pharmaceuticals) and nutritional supplements. The oral bioavailability in humans is low (estimated < 10%) but increases up to 4-fold when taken with food (Taylor et al. 2019). Similar results were reported in rats (Zgair 2016). CBD, a highly lipophilic molecule (log *P* > 6.3), is involved in lymphatic transport. The relative bioavailability via lymph system was 13% when administered in a long chain triglycerides-free nanoemulsion (Rysanek et al. 2025), 39% in a sunflower oil-based microemulsion (Jelinek 2022), and 50% in a simple sunflower oil solution (Rysanek et al. 2025).

The aim of this study was to develop a robust animal model for lymphatic transport testing in conscious pigs and to measure the lymphatic transport and other pharmacokinetic parameters of CBD. The generated data were further used to provide an interspecies comparison between pigs and rats.

## Materials and methods

### Animals

Young adult landrace pigs of both sexes (age 5–6 months, bodyweight 50–70 kg) were used. The pigs were purchased from the Institute of Animal Science, Prague, Czech Republic. The acclimation period before the experiment lasted at least one week. The animal experiments were carried out at the Institute of Physiology, First Faculty of Medicine, Charles University in Prague, in the period between February 2021 and May 2024.

### Anesthesia

The pigs had free access to water and pellet food. First, they were sedated with ~20 mg of midazolam and ~1200 mg of ketamine administered i.m. into the neck. They were transported from the pen into the operating theater and placed on the operating table while still maintaining spontaneous breathing. A proper monitoring of the vital functions was started (peripheral oxygen saturation, ECG, body temperature). A vascular access (20 G cannula) was placed into the ear vein. A total intravenous anesthesia was initiated using propofol at ~10 mg/kg/h, midazolam at 0.3 mg/kg/h, and morphine at 0.25 mg/kg/h. The pigs were hydrated with Ringer solution (500 ml/h) throughout the entire period of anesthesia. A bolus of 50–100 mg of propofol was administered, and endotracheal intubation was performed using a large adult Miller laryngoscope blade and an 8.0 ID Magill endotracheal tube. Mechanical ventilation was started using a pressure-controlled mode with FiO_2_ 35% and a PEEP of 5 mbar. The respiratory rate and inspiration pressure were adjusted to maintain an end-tidal CO_2_ partial pressure of ~40 mm Hg. A proper long-term vascular access was secured on the ear of the opposite side. A 4 Fr peripherally inserted central venous catheter (Lifecath CT PICC easy, Vygon, Czech Republic) was inserted through the ear vein down into the vena cava superior using a Seldinger technique. Its correct positioning was checked on the chest X-ray. The PICC catheter was further used for i.v. drug administration during the surgery and in the postoperative phase and also for the repetitive blood sampling later during the lymphatic transport study.

### Surgery

**Figure 1.**
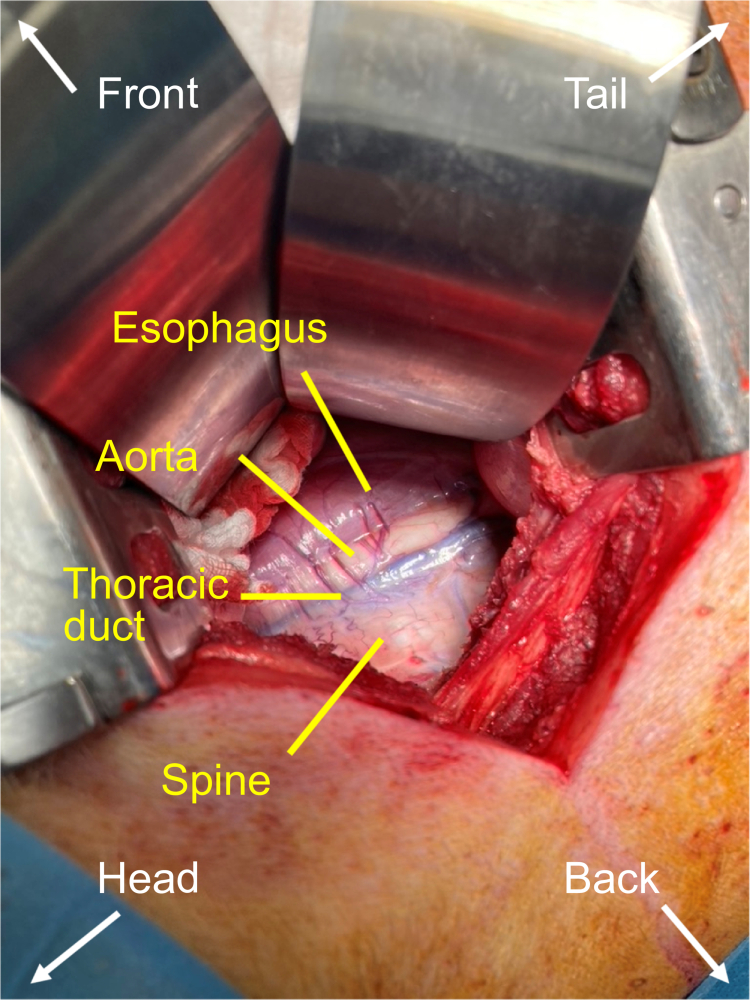
Topographic anatomy of the thoracic duct. The thorax was opened in the fourth intercostal space on the right side. The lung was pushed aside.

The pigs were positioned on the left side, and the right flank was prepared for right-sided thoracotomy in the fourth or fifth intercostal space. Cefazolin (1 g) and rocuronium (50 mg) were administered shortly before the cut to prevent perioperative infection, and to achieve the necessary muscle relaxation, respectively. The thoracic duct was found in the groove between the spine and aorta in the close proximity to esophagus (Figure 1).

**Figure 2.**
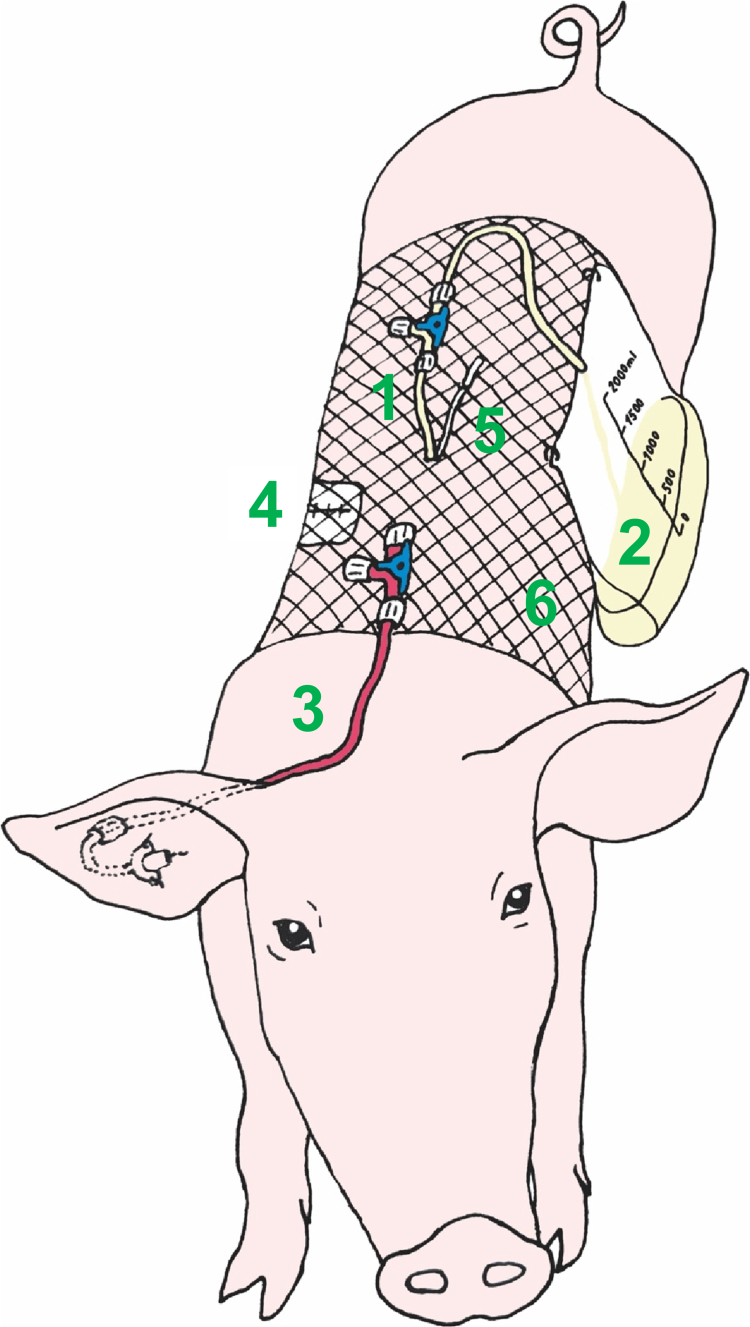
Thoracic lymph duct-cannulated conscious pig model in overview. (1) The thoracic lymph duct catheter was tunneled through the skin on the back of the animal and extended with a three-way stop-cock allowing easy catheter flushing and controlling its patency. (2) The modified urinary bag was used for the lymph collection. (3) The blood catheter, a peripherally inserted central venous catheter (PICC) was extended with a three-way stop-cock used for repetitive blood sampling and intravenous drug administration. (4) Surgical wound after right-sided thoracotomy. (5) Tiny local anesthetics catheter with its tip placed in the surgical wound, tunneled through the skin at the same spot like the lymph catheter and used for the postoperative analgesia. (6) Net elastic bandage used to secure the catheters and the lymph bag. The sterile covers of the blood catheter on the ear and the lymph catheter on the back are not shown in the figure to increase the clarity.

The thoracic duct was first cannulated using a 20 G cannula. A guide wire was then inserted into the duct, and the cannula was removed (Seldinger technique). Finally, a 5 Fr peripherally inserted central venous catheter (Lifecath CT PICC easy, Vygon, Czech Republic, catalogue no. V021292215) was inserted deeply into the duct (15–20 cm), with the tip positioned approximately at the level of the diaphragm. The catheter was fixed in place with two to three drops of tissue adhesive (Histoacryl, B. Braun Surgical, Spain) and two sutures. It was then tunneled through the intercostal muscles and skin towards the back of the animal. The same tunneling canal was used for the placement of the local anesthetics catheter (BTPE-50 tubing with a PinPort, OD 0.97 mm, length ~30 cm; Instech Laboratories, USA). This small catheter was used later after surgery for intermittent local anesthetics dosing directly into the thorax wall for analgesia. The first dose of local anesthetic (bupivacaine 50 mg in 20 ml) was applied using a thin needle into the surgical wound margins shortly before the wound closure. The thorax wall was closed using a thick polydioxanone suture (ribs and intercostal muscles) and a skin stapler. The lymph catheter was connected to a urinary bag for lymph collection over a three-way stop-cock enabling easy catheter flushing and maintenance. The surgical wound and the catheter entry points were covered with sterile bandages. The whole trunk of the animal was slipped into an elastic bandage for fixation, coverage and protection of the catheters (lymph catheter, local anesthetics catheter and later, after i.v. infusion disconnection, the central venous catheter, which was extended with a line that ran from the ear over the neck towards the back of the animal where all three catheters were easily accessible; see Figure 2). The lymph collection bag was attached to the elastic bandage and hung on the side of the animal.

### Postoperative care

Shortly before the end of the surgery, 0.4  mg/kg of meloxicam was administered i.m. in order to secure a basic long-term analgesia. At the same time, morphine 10–20 mg i.v. bolus was administered. Dexmedetomidine 1.2 μg/kg/h infusion was started as light sedation for the postoperative phase. The continuous propofol and morphine infusions were stopped. Midazolam was stopped 1 h after its initiation because of its longer biological half-life. After regaining spontaneous breathing, the pigs were extubated and transported into monitored pens.

The recovery period took 18–22 h (until the tested drug formulation dosing on the next day morning). The analgesic protocol included morphine, paracetamol, and bupivacaine. Morphine 10  mg i.v. was administered every 6 h + boluses 10–20  mg whenever necessary but not less than 8 h before the tested drug formulation was dosed to avoid any negative opioid effects on the intestinal peristalsis during the study. The paracetamol 1 g was administered i.v. every 8 h together with cefazolin 1 g in a combined short infusion during the whole recovery period. Bupivacaine 50 mg was administered locally every 6 h through the local anesthetics catheter.

The pigs were monitored closely by a trained anesthesiologist during the whole recovery period. They received oxygen via an oxygen mask, and peripheral oxygen saturation was measured during the first one or two postoperative hours. The pigs gradually woke up and were usually able to stand up within several hours after surgery. The dexmedetomidine infusion was disconnected. The pigs had unrestricted access to water and were fed one standard portion of pellet food in the evening.

A special attention was paid to the lymph catheter. The lymph flow was monitored closely, and the catheter was flushed with 1–2 ml of saline every 15 min. When the lymph flow was stable and the catheter was patent, the flushing periods were extended to 30 or 60 min (and up to 180 min at night). The lymph bag was emptied regularly, and the amount of lymph was recorded. In case of abrupt catheter patency loss, the catheter was flushed with alteplase (fibrinolytic agent) 2.5 mg dissolved in 2.5 ml of water for injection. The alteplase solution was administered into the lymph catheter and the three-way stop-cock was closed for 5 min to provide enough time for the blood clots to dissolve. After that, the stop-cock was opened again, and the lymph catheter was flushed repeatedly with normal saline.

### Drug formulations

CBD was administered orally in two distinct formulations, a nanoemulsion and a sesame oil-based formulation. The nanoemulsion used in this study was based on the optimized composition previously developed and reported (Rysanek et al. 2025). In the present study, this optimized formulation was employed without further modification. Briefly, CBD (Pharmabinoid, Netherlands) was mixed with 1 g of propylene glycol monocaprylate (Gattefossé, France), 2.5 g of Kolliphor® EL (BASF, Germany), and 1.5 g of diethylene glycol monoethyl ether (Gattefossé, France) to form the organic phase. Prior to administration, the organic phase was mixed with 10 ml of water to produce the nanoemulsion. One oral dose of the nanoemulsion contained 200 mg of CBD in 15 ml. The nanoemulsion was also used for intravenous dosing in one animal (20 mg of CBD in 1.5 ml). The sesame oil-based formulation was the original registered drug product used in humans (Epidyolex®, Jazz Pharmaceuticals, 100 mg/ml, administered dose: 200 mg of CBD in 2 ml of solution).

### Lymphatic transport study

**Figure 3.**
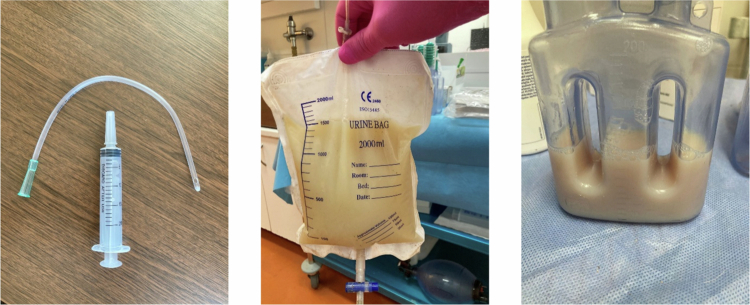
(Left) A 20 ml syringe with a rigid plastic tube used for the oral drug administration deep into the throat. (Middle) A urinary bag with a large amount of porcine lymph collected overnight. (Right) A 200 ml Redon bottle with a 1-h lymph sample.

An overview of the thoracic lymph duct cannulated conscious pig model is shown in Figure 2. A two-period, cross-over pharmacokinetic study with lymphatic transport measurements was conducted. After an overnight recovery from the surgery and at least 10 h of fasting (although unrestricted access to water), the pigs were dosed with 200 mg of CBD in the form of an oil solution or a nanoemulsion. The animals were put under mild anesthesia using a propofol bolus (~100 mg i.v.). They were pulled in an upright position, the mouth was opened, and the drug formulation was instilled deep into the throat using a syringe extended with a rigid plastic tube (Figure 3). The pigs were then held in the upright position for another several minutes, and the neck was rubbed to facilitate the swallowing and prevent any regurgitation of the dosed drug. After that, the mouth was opened again and checked for any unswallowed drug formulation before laying the pig on the floor to allow it to recover from the sedation. The pigs were fasted for 4 h after the dosing and subsequently received a standard dose of pellet food twice a day.

After drug administration, the lymph was continuously collected. The urinary bag that was used for the lymph collection overnight was replaced with a 200 ml Redon bottle where a mild negative pressure was maintained by removing 20 ml of air twice an hour to facilitate the lymph flow (Figure 3). The Redon bottle was emptied every hour. The lymph volume was measured and a 2 ml aliquot was taken for CBD concentration analysis. Blood (2 ml) was sampled from the ear catheter at the same time points as the lymph. The whole sampling protocol consisted of 13 lymph aliquots and blood samples that were taken at 1, 2, 3, 4, 5, 6, 7, 8, 9, 10, 11, 12, and 24 h post-dose. After the samples were collected at 12 h, the Redon bottle was replaced with a urinary bag and the lymph was collected overnight. After the last sampling (24 h post-dose), there was a one-day wash-out period, and the second period of dosing and sampling followed.

One pig was cannulated as previously described but was not orally dosed. Instead, it was dosed intravenously to provide an intravenous CBD pharmacokinetic profile for the calculation of the total absolute bioavailability of CBD in the orally dosed animals (see below). A 1.5  ml nanoemulsion containing 20  mg of CBD was diluted with water for injection to 5 ml and administered through the ear catheter. After thorough flushing with saline and repeated blood aspiration to remove any possible drug contamination from the catheter, the blood sampling followed. The blood (2 ml) was taken at 5, 15, and 30 min and at 1, 1.5, 2, 2.5, 3, 4, 6, 8, 12, and 24 h. The lymph was not sampled in the intravenously dosed animal.

Blood samples were centrifuged (4500 rpm for 5 min) and the serum was extracted and stored in −80 °C until analysis. The lymph sample aliquots were stored in −80 °C until analysis, with no further adjustment. The laboratory was unaware of animal assignment to a particular experimental group (laboratory blinding).

### Bioanalysis

Quantification of CBD in pig serum and lymph samples was performed using a previously described UHPLC-MS/MS (ultra-high-performance liquid chromatography–tandem mass spectrometry) method with an isotopically labeled internal standard, as detailed in our earlier work (Jelinek 2022). Briefly, proteins in the serum and lymph matrices were precipitated using acetonitrile. Specifically, 20 µL of sample was mixed with 80 µL of pure acetonitrile containing cannabidiol-d3 (30.0 ng/mL) as the internal standard. The mixture was vortexed thoroughly and centrifuged at 10,000 × g for 8 min. Subsequently, 60 µL of the supernatant was transferred to an LC vial for analysis. UHPLC‒MS/MS analyses were conducted using a Shimadzu Nexera X3 ultrahigh-performance liquid chromatograph coupled with a Shimadzu Triple Quad 8045 mass spectrometer (Kyoto, Japan). Chromatographic separation was achieved on a Poroshell 120 EC-C18 column (50 × 2.1 mm, 1.9 µm; Agilent Technologies, Santa Clara, CA, USA). The mobile phase consisted of solvent A (0.1% formic acid in deionized water) and solvent B (methanol with 0.1% formic acid). The flow rate was 0.4 mL/min, with an injection volume of 2 µL. The column temperature was maintained at 40 °C, and the sample vials were kept at 10 °C. The gradient elution program (time [min]/%B) was as follows: 0.0/50, 0.5/50, 2.5/90, 3.5/90, 4.0/50, and 5.5/50. Detection was performed in positive electrospray ionization (ESI) mode. The ion source parameters were set as follows: nebulizing gas flow, 3 L/min; heating gas flow, 10 L/min; interface temperature, 300 °C; desolvation line temperature, 250 °C; heat block temperature, 400 °C; and drying gas flow, 10 L/min. Data acquisition was carried out in multiple reaction monitoring (MRM) mode. The monitored transitions were m/z 315.2 → 193.1 for cannabidiol (Q1 pre-bias: –16 V; Q3 pre-bias: –20 V; collision energy: –22 V) and m/z 318.2 → 196.1 for the deuterated internal standard cannabidiol-d3 (Q1 pre-bias: –16 V; Q3 pre-bias: –22 V; collision energy: –35 V). Calibration curves were constructed individually for blank serum and lymph matrices by spiking with seven concentrations of the analyte. The response was calculated as the ratio of the analyte peak area to that of the internal standard and plotted against the cannabinoid concentration. A weighted least-squares linear regression model with a 1/x² weighting factor was applied to improve accuracy at low concentrations. Method validation was performed in accordance with the European Medicines Agency (EMA) Guideline on bioanalytical method validation (EMA, 2022). The assay demonstrated excellent linearity over the range of 1–1000 ng/mL (R² ≥ 0.9992), with a lower limit of quantification (LLOQ) of 1 ng/mL. The intra-day and inter-day precision values were  ≤10.1%, and the accuracy was within  ±7.3%. Analyte recovery ranged from 97.2% to 103.1%. All the validation parameters complied with the EMA guideline criteria, confirming that the method is suitable for its intended use.

### Data analysis and statistics

The area under the curve (AUC) values for serum and lymph nodes were determined using linear trapezoidal rule. The exact actual sampling times were used for this purpose. Scheduled sampling times were used for mean concentration plotting in the graphs. The PK-solver add-on for MS Excel was used for all basic pharmacokinetic calculations (Zhang et al. 2010). GraphPad Prism version 10.5.0 (GraphPad Software, San Diego, CA, USA) was used for all the statistical analyses and graph plotting. A paired t-test was used to compare pharmacokinetic and lymphatic transport parameters between the drug formulations and an unpaired t-test was used for the interspecies comparisons. The level of significance was set to *p* < 0.05.

### Calculation of lymphatic transport parameters

Lymphatic transport parameters were defined and calculated as previously described in the experiments with rats (Rysanek et al. 2020; Rysanek et al. 2021; Pozniak et al. 2024; Rysanek et al. 2025). Briefly, absolute bioavailability via lymph (F_AL_) was defined as the percentage of the administered drug dose absorbed into the lymph. It was determined directly from the lymph volume and drug concentration. Absolute bioavailability via the portal vein (F_AP_) was analogically defined as the percentage of the administered drug dose reaching the systemic circulation after direct absorption into the blood. It was calculated using equation:

(1) $$F_{\text{AP}}\;={\mathrm{AUC}}_{\text{ent}}\;/\;{\text{AUC}}_{\mathrm{iv}}$$

where AUC_ent_ is the area under the dose-normalized blood concentration‒time curve after enteral dosing in lymph duct cannulated (i.e. lymph deprived) animals, and AUC_iv_ is the respective parameter in a separate intravenously dosed animal. The total absolute bioavailability (F) in the lymph duct of cannulated animals was calculated as follows:

(2) $$F=\;{\text{F}}_{\mathrm{AL}}+F_{A\text{P}}$$

Relative bioavailability via lymph (F_RL_) was defined as the percentage of systemically available drug that was absorbed via the lymph. It was calculated using the following equation:

(3) $$F_{\mathrm{RL}}=F_{\mathrm{AL}}\;/\;F$$

## Results

### CBD pharmacokinetics and lymphatic transport in pigs

**Figure 4.**
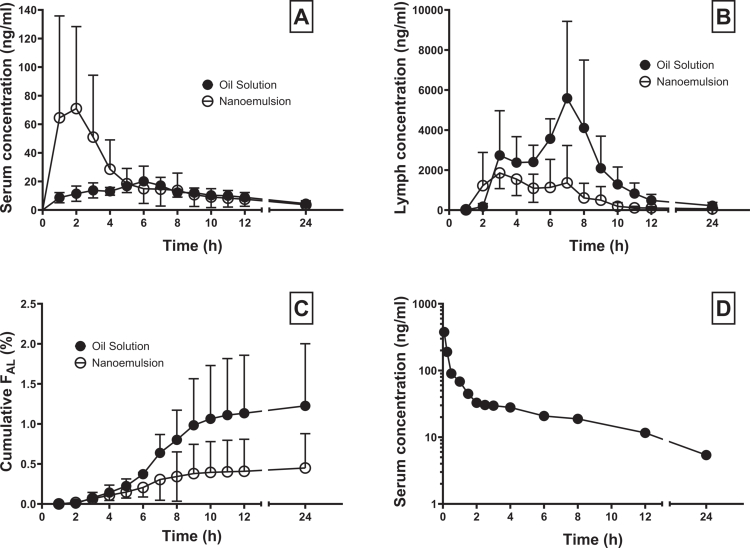
(A) Mean ± SD CBD serum pharmacokinetic profiles, (B) lymph pharmacokinetic profiles, and (C) cumulative lymphatic transport profiles after oral administration of CBD 200 mg in the form of a sesame oil-based solution (Epidyolex, 2 ml) and a nanoemulsion (15 ml) to three thoracic lymph duct-cannulated pigs. (D) CBD serum pharmacokinetic profile after intravenous administration of CBD 20 mg in the form of a nanoemulsion (1.5 ml) to one pig. F_AL_ – absolute bioavailability via lymph.

The full two-period lymphatic transport study was successfully completed in three pigs. The CBD serum and lymph concentrations and cumulative lymphatic transport, as well as an intravenous pharmacokinetic profile are shown in Figure 4. The pharmacokinetic and lymphatic transport parameters are summarized in Tables 1 and 2.

**Table 1.** Mean ± SD CBD lymphatic transport parameters after oral administration of CBD 200 mg in the form of a sesame oil-based solution (Epidyolex, 2 ml) and nanoemulsion (15 ml) to three thoracic lymph duct-cannulated pigs.

Formulation	C_max_ lymph (ng/ml)	C_max_ serum (ng/ml)	C_max_ lymph/serum ratio	T_max_ lymph (h)	T_max_ serum (h)	T_max_ lymph/serum ratio	
Oil solution	6613 ± 2220	22.1 ± 7.5	364 ± 223	5.68 ± 1.89	4.96 ± 1.42	1.12 ± 0.08	
Nanoemulsion	2843 ± 702	81.6 ± 56.6	184 ± 231*	4.04 ± 2.14	2.67 ± 1.70	1.67 ± 0.30	
	AUC_inf_ lymph (ng*h/ml)	AUC_inf_ serum (ng*h/ml)	AUC_inf_ lymph/serum ratio	F (%)	F_AP_ (%)	F_AL_ (%)	F_RL_ (%)
Oil solution	34488 ± 10319	317 ± 89	123 ± 66	6.1 ± 0.9	8.7 ± 6.7	0.5 ± 0.3	20 ± 10
Nanoemulsion	11281 ± 5157*	591 ± 488	9.6 ± 59*	9.2 ± 6.6	4.9 ± 0.9	1.2 ± 0.6	11 ± 13

F – total absolute bioavailability, F_AP_ – absolute bioavailability via portal vein, F_AL_ – absolute bioavailability via lymph, F_RL_ – relative bioavailability via lymph, *marks a significant difference between the formulations in paired t-test (*p *< 0.05).

**Table 2.** CBD serum pharmacokinetic parameters after intravenous administration of CBD 20 mg in the form of a nanoemulsion (1.5 ml) to one pig.

C_max_ (ng/ml)	376.6	V_ss_ (l)	312
AUC_inf_ (ng*h/ml)	594.4	V_ss_ (l/kg)	4.88
T_1/2_ (h)	8.52	CL (l/h)	33.6
		CL (l/h*kg)	0.53

Vss – steady-state volume of distribution, CL – clearance.

The amount of CBD absorbed directly into the systemic circulation was rather low ( < 10%) and did not differ significantly between the formulations. There was no statistically significant difference in the serum C_max_, serum AUC_inf_ or absolute bioavailability via the portal vein (F_AP_). The CBD was absorbed at moderate to slow speed with the serum T_max_ reaching 3–5 h.

A large amount of CBD was absorbed into the lymph. The mean total CBD exposure in the lymph fluid, expressed as AUC_inf_, was 10-times higher compared to serum after the nanoemulsion administration and 123-times higher after the oil solution administration. The oil solution stimulated the CBD lymphatic transport more than the nanoemulsion. The lymphatic AUC_inf_, and the AUC_inf_ and C_max_ lymph/serum ratios were significantly higher for the oil solution compared to nanoemulsion. The differences in the absolute bioavailability via lymph (F_AL_) and the relative bioavailability via lymph (F_RL_) did not reach the statistical significance in the limited number of individuals used in the study, but a trend was clearly visible (*p* = 0.06 for both).

### Interspecies comparison

Recently, CBD lymphatic transport parameters were assessed in anesthetized, lymph duct-cannulated rats (Rysanek et al. 2025). Table 3 provides an interspecies comparison between the conscious pigs and the anesthetized rats. For the oil solution, there was only a significant difference in the relative bioavailability via lymph (F_RL_), which was lower in pigs (*p* = 0.01), though it was still considerably high. For the nanoemulsion, there was a lower total absolute bioavailability (F) and bioavailability via lymph (F_AL_) in pigs; *p* = 0.03 and 0.01, respectively. The difference in the bioavailability via portal vein narrowly missed the preset level of statistical significance (*p* = 0.052). On the other hand, the relative bioavailability via the lymph system was almost identical in pigs and rats after the nanoemulsion administration.

**Table 3.** Mean ± SD lymphatic transport parameters interspecies comparison between pigs and rats. The thoracic lymph-duct cannulated conscious pigs were dosed with CBD 200 mg (~3.5 mg/kg) orally in the form of sesame oil-based formulation (Epidyolex) and a nanoemulsion. The anesthetized mesenteric lymph duct-cannulated rats were dosed with CBD 10 mg (~20 mg/kg) intraduodenally in the form of a pure sunflower oil and a nanoemulsion. The data for rats are taken from Rysanek et al. (2025). The data for pigs and rats are both calculated 8 hours after the dosing.

Formulation	Species	N	F (%)	F_AP_ (%)	F_AL_ (%)	F_RL_ (%)
Oil solution	Pigs	3	3.6 ± 0.2	2.8 ± 0.2	0.80 ± 0.30	22 ± 8*
	Rats	6	4.0 ± 1.5	1.9 ± 0.9	2.0 ± 0.9	50 ± 12
Nanoemulsion	Pigs	3	7.0 ± 4.0*	6.6 ± 4.2	0.34 ± 0.25^*^	13 ± 16
	Rats	6	24 ± 9	21 ± 9	2.6 ± 1.0	13 ± 8

* marks a significant difference between the species in unpaired t-test (*p* < 0.05).

### Animal model development

In the late phase of the animal model development, twelve pigs were utilized. Slight methodology modifications were made that led to the finalization of the experimental protocol as described in the methods section of this article. The thoracic duct cannulation was successful in 11 animals (92%). The first period of dosing and sampling was completed in 9 animals (75%). The full two-period, cross-over study was completed in 3 animals (25%), the results from which are reported above. The mean lymph flow in the successfully completed dosing and sampling periods was between 36 and 109 ml/h. The predominant reason for a premature experiment termination was a loss of lymphatic catheter patency due to blood clot formation in or around the catheter tip.

The thoracic duct in the first several pigs was cannulated using a large (5 Fr inner diameter) reinforced catheter with a guiding sheath and hydrophilic coating (Destination, Terumo Corporation, Japan, catalogue no. GS*F5ST1C45). The insertion depth was ~5 cm. The catheter was flushed frequently with heparinized saline (20  IU/ml), and in case of patency loss, concentrated heparin (5000 IU/ml, volume 1–2 ml) was applied, followed by alteplase (2.5 mg in 2.5 ml) if necessary. However, the combination of a large and rather rigid catheter placed in the lymph duct and a pro-active local anticoagulant and thrombolytic therapy led to a high risk of bleeding into the lymph and a high rate of catheter patency loss due to blood clot formation.

Therefore, the experimental protocol was changed, and a much softer and thinner (5 Fr outer diameter) polyurethane lymphatic catheter (Lifecath CT PICC easy, Vygon, Czech Republic, catalogue no. V021292215) was used in the last few pigs. The catheter was inserted deeper into the lymphatic duct (~20 cm) to possibly avoid any blood contribution from the lympho-venous junctions present in the thorax (Cope 1995). Furthermore, the catheter was flushed with normal saline, no concentrated heparin was used, and alteplase was used only as a method of last resort in case of patency loss. These changes reduce the amount of blood mixed into the lymph system and increase the overall success rate.

## Discussion

### CBD pharmacokinetics and lymphatic transport in pigs

CBD total absolute oral bioavailability assessed in the conscious thoracic lymph duct-cannulated pig model was low (<10%) regardless of the formulation administered, which corresponds to the low oral bioavailability anticipated in men (WHO, 2018). The CBD volume of distribution of ~4.5 l/kg measured after intravenous dosing corresponds to the range of 2.5–10 l/kg reported for men (Qian et al. 2024), whereas the half-life of ~8.5 h is shorter compared to humans (~24  h) (Ohlsson et al. 1986).

The lymphatic transport plays an important role in the general pharmacokinetics of CBD since 10%–20% of systemically available drugs were transported through the intestinal lymphatic system. The oil solution stimulated the lymphatic transport slightly more than the nanoemulsion. This fact can be easily explained by the absence of long-chain triglycerides in the nanoemulsion formulation that are known to increase the lymphatic transport by providing the material for the assembly of intestinal lipoprotein particles (chylomicrons) (Caliph et al. 2000).

### Interspecies comparison

In the literature, it was reported that for the compound halofantrine, lymphatic transport (both absolute and relative) increases with increasing bodyweight of the animal (dog > rat > mouse) (Trevaskis et al. 2013, 2020). This finding is not supported in our study for the molecule of cannabidiol. Regarding the oil solution, there is no significant difference in the absolute bioavailability via lymph (F_AL_) between the pigs and the rats. The mean ± SD relative bioavailability via lymph (F_RL_) was even significantly lower in pigs than in rats (22 ± 8% vs. 50 ± 12%, *p* = 0.01). For the nanoemulsion, the F_RL_ was similar in both species, but the F_AL_ was lower in pigs than in rats (0.34 ± 0.25% vs. 2.6 ± 1.0%, *p* = 0.01).

The oil solutions administered to pigs and rats differed. The pigs received the registered original CBD drug product (Epidyolex), which consists of ~90% purified sesame oil. The rats received a pure sunflower oil. Both oils contain a large amount (>80%) (Akkaya 2018) of long-chain (C ≥ 16) triglycerides that are known to stimulate the lymphatic transport. However, the relative amount of lipids administered to pigs was significantly lower compared to rats (2  ml of drug formulation representing ~0.03 ml/kg in pigs *vs.* 1  ml of drug formulation representing ~2.5 ml/kg in rats). Moreover, the pigs were fasted before the drug administration, while the rats were not and received additionally 1 ml of olive oil before the cannulation surgery to make the mesenteric lymph duct visible.

The nanoemulsion was long chain triglycerides-free and had the same composition for both pigs and rats, with the exception of a slightly different CBD concentration. Moreover, the difference in the relative amount of drug formulation administered to both species was smaller than that in the case of the oil solution (15 ml of drug formulation representing ~0.25 ml/kg in pigs vs. 1 ml of drug formulation representing 2.5 ml/kg in rats). The absence of long-chain triglycerides is probably responsible for the almost identical CBD partitioning into the lymph (F_RL_) in both species. The differences in F, F_AP_, and F_AL_, which are all lower in pigs than in rats, could be explained by lower CBD solubilization in the gastrointestinal tract (fasted pigs *vs.* fed rats), resulting in lower intestinal absorption. All these findings suggest that the rule of increasing F_AL_ and F_RL_ with increasing bodyweight of the animal, as described for halofantrine could still be valid also for CBD, but a study with a standardized amount of lipids administered (in mg/kg bodyweight) would be necessary to prove it.

### Animal model development

The development of the conscious thoracic lymph duct-cannulated pig model was a challenging task. Initially, thoracic duct cannulation on the neck (i.e. without thoracotomy) was sought as previously described in several studies with dogs (Khoo et al. 2001; Shackleford et al. 2003; White et al. 2009) and one study with pigs (Yen and Davies 2016). However, this technique was not feasible since the thoracic duct was not easily accessible and was not recognizable from the surrounding tissues. Therefore, the decision to access the lymph duct in the thorax was made as previously described in some other surviving conscious pig models (Jensen et al. 1990; Mendenhall and Horvath, 1996 ; Chanoit et al. 2007). The fears of a difficult postoperative recovery due to pain and a risk of pneumothorax were not confirmed. With combined local and systemic analgesia, the pigs were able to stand up and eat just several hours after the surgery and there was not a single case of pneumothorax.

Many drugs were administered in the postoperative phase to help the pigs recover. These factors could potentially interact with the CBD pharmacokinetics on the next day. Morphine is known to slow down the intestinal peristalsis which could interfere with the CBD intestinal absorption. Therefore, the last morphine i.v. dose was given not less than 8 h before the CBD administration. Other drugs (meloxicam, paracetamol and bupivacaine) are not expected to interact with the CBD absorption based on their mechanisms of action. An interaction at the level of CBD metabolism is unlikely due to largely different metabolic pathways of all the compounds.

The single biggest problem of the whole experimental procedure was the mixing of blood into the lymph, which led to blood clot formation in the thoracic duct around the catheter tip and in the tubing system and the loss of catheter patency. The source of blood was presumably either the thoracic duct wall and surrounding tissues injured by the process of cannulation or persistent lympho-venous junctions in the thorax or elsewhere in the peripheral lymphatic system (Cope 1995). A particular source of blood could not have been easily distinguished in every animal. In some cases, the cannulation was difficult and traumatic, with bleeding around the duct observed during the surgery. In other animals, the cannulation was fully atraumatic, but the blood started mixing into the lymph later. Generally, the amount of blood in the lymph varied throughout the hours and days of the experiment. The formation of blood clots in the lymphatic catheter has been reported as a problem in at least one other study describing chronic lymph duct cannulation in pigs (Jensen et al. 1990).

Although problematic regarding the lymph catheter patency, the blood in the lymph system was not seen as a factor that dramatically affecting the measured lymphatic transport parameters since the CBD concentrations in the systemic blood were generally two orders of magnitude lower than the lymph concentrations (see Figure 4). Moreover, the amount of blood in the sampled lymph as assessed using a hematocrit measurement was low (<10%). The refining of the experimental protocol in the final phase of the development reduced the risk of bleeding and catheter patency loss. These steps included: (1) the use of a soft polyurethane catheter to prevent duct wall injury, (2) catheter insertion deeper into the duct (~20 cm) to exclude any possible lympho-venous junctions in the thorax, and (3) catheter flushing with normal saline with no heparin to prevent possible bleeding from the duct wall or elsewhere in the lymphatic system to stop.

This was a pilot study including data from three pigs for CBD lymphatic transport assessment. Only animals with a completed two-period cross-over study protocol were used in order to exploit the advantages of the reduced effect of the interindividual variability on the test results. In the future, larger numbers of animals can be used to test CBD or other compounds.

The thoracic lymph duct cannulation and lymph sampling in conscious pigs is a challenging but feasible method for measuring lymphatic drug transport in higher species, with a good possibility of data extrapolation to human. The pharmacokinetic testing of CBD, particularly in pigs, is of great interest and importance because of its extensive metabolism via the CYP2C9 enzyme, which is expressed in pigs but is not expressed in dogs (Dalgaard 2015; Qian et al. 2024). In the future, the pig model can be used for the testing of standard human-sized drug formulations and testing of food effect on the lymphatic drug transport, in both cases applying a cross-over study design that reduces the negative effect of high interindividual variability.

## Conclusions

A large portion of CBD is transported through the mesenteric lymphatic system after oral administration in pigs. The oil solution used as a drug formulation stimulates the lymphatic transport more than a nanoemulsion devoid of long chain triglycerides. An interspecies comparison was made between pigs and rats using recently published data. While the oil solution achieved a higher relative bioavailability via lymph in rats than in pigs, the same parameter was almost identical for the nanoemulsion. The thoracic lymph duct-cannulated conscious pig model is a challenging but feasible method for lymphatic drug transport assessment.

## Supplementary Material

Supplementary material ARRIVE Author Checklist

## Data Availability

The data supporting this work are accessible upon reasonable request from the corresponding author.
